# Supplementary material for: rasterdiv—An Information Theory tailored R package for measuring ecosystem heterogeneity from space: To the origin and back
Source: Methods Ecol Evol. 2021 May 3;12(6):1093–102. doi: 10.1111/2041-210X.13583 (PMC8252722; doi:10.1111/2041-210X.13583)
Supplement: Supplementary file 1 — Appendix1 [file MEE3-12-1093-s001.pdf]

Appendix 1
Mathematical proof of the equivalence between Rényi entropy and Shannon's H given $\alpha \to 1$

`rasterdiv` - an Information Theory tailored R package for measuring ecosystem heterogeneity from space: to the origin and back

December 12, 2020

Theorem (De l'Hôpital). *Let $f, g : (a, b) \mapsto \mathbb{R}$ be two functions such that*

- $\lim_{x\to a} f(x) = \lim_{x\to a} g(x) = 0$
- *f and g are derivable in (a, b) with $g'(x) \neq 0$ for every $x \in (a, b)$*
- *the limit $\lim_{x\to a} \frac{f'(x)}{g'(x)} = L$ with $L \in \mathbb{R}$*

then

$$\lim_{x\to a} \frac{f(x)}{g(x)} = L.$$

We now verify if the hypothesis hold in our case. Let $f : (1, +\infty) \mapsto \mathbb{R}$ and $g : (1, +\infty) \mapsto \mathbb{R}$ be functions such that $f(x) = \log(\sum_{i=1}^{N} p_i^x)$ and $g(x) = 1 - x$ for every $x \in (1, +\infty)$. Since p_i is a fixed number in $(0, 1)$ for every $i \in \{1, \ldots, \}$ with $\sum_{i=1}^{N} p_i = 1$, then

$$f(1) = \log(\sum_{i=1}^{N} p_i) = 0.$$

Moreover, since f is a composition of derivable functions in $(1, +\infty)$, f is derivable in the definition domain. It is trivial that $g(1) = 0$, that g is

derivable in the domain set and that $g'(x) \neq 0$ for every $x \in (1, +\infty)$. The limit of the derivatives ratio is:

$$\lim_{x \to 1} \frac{f'(x)}{g'(x)} = \lim_{x \to 1} \frac{(\sum_{i=1}^N p_i^x)^{-1} (\sum_{i=1}^N p_i^x \log p_i)}{1} = \sum_{i=1}^N p_i \log p_i$$

which is finite due to the hypothesis over p_i. Since all the hypotheses of De l'Hôpital's Theorem hold, the thesis follows, i.e.

$$\lim_{\alpha \to 1} H_\alpha = H,$$

or Rényi 's entropy equals to Shannon's H for $\alpha \to 1$.
